# Local W-like Haplotype Architecture in the Candidate Sex-Determining Region of Black Tiger Shrimp

**DOI:** 10.3390/biology15211962

**Published:** 2026-11-01

**Authors:** Yangyang Ding, Yutong Zhao, Pengying Li, Song Jiang, Yundong Li, Qibin Yang, Ziyi Jiang, Lishi Yang, Jianhua Huang, Jianzhi Shi, Yafei Duan, Falin Zhou

**Affiliations:** 1 Key Laboratory of South China Sea Fishery Resources Exploitation and Utilization, Ministry of Agriculture and Rural Affairs, South China Sea Fisheries Research Institute, Chinese Academy of Fishery Sciences, Guangzhou 510300, China; dingyangyang93@163.com (Y.D.);; 2 Southern Marine Science and Engineering Guangdong Laboratory (Zhuhai), Zhuhai 519082, China; 3 Sanya Tropical Fisheries Research Institute, Sanya 572018, China

**Keywords:** black tiger shrimp (*Penaeus monodon*), homomorphic sex chromosomes, read-backed phasing, structural variation, haplotype architecture

## Abstract

While female heterogamety in black tiger shrimp (*Penaeus monodon*) is consistent with a ZW/ZZ system, the sequence organization of its sex-associated region remains unclear. In this study, we examined the candidate sex-determining region on chromosome 19 (2.71–2.89 Mb) using PacBio HiFi reads from a marker-supported ZW female, genotypes from 200 full-sib offspring, local assembly, and public long reads. Three phase blocks overlapped this interval. Sex-associated SNPs assigned W-like and Z-like alleles in two blocks, while the central block remained unoriented. The first block contained gene24, a partial *dmd-3*-related gene model, and a 54 bp insertion in gene25 intron 1 (INS00000105; INS54). This insertion, supported by 17 W-like HiFi reads and two local assemblers, was called in 100/100 female offspring and 1/99 called males; one male was uncalled. Sanger reads confirmed the insertion and both flanks in three selected market-sourced females and the no-insertion junction in three selected males. Public reads from a Thai female reproduced the insertion-bearing structure, whereas Australian male reads supported the alternative structure. Separate Thai and Chinese long reads crossed the two phase gaps in their respective samples. Together, these results define local female-associated sequence differences and targets for functional investigation, rather than complete W and Z chromosome sequences.

## Simple Summary

The sex-determining system of black tiger shrimp is usually represented as ZW females and ZZ males. While the two chromosome copies look similar, their DNA may differ within the region associated with sex. In this study, we examined this region using long DNA reads from a female shrimp and genotypes from 200 offspring, and we identified three separately phased segments, meaning that variants could be linked within each segment but not between them. Sex-associated markers identified the female-associated sequence in two segments: one contained a 54-base insertion that was supported by the female’s reads and local assemblies. This insertion occurred in all female offspring and one called male, while Sanger sequencing also detected it in three selected market-sourced females, with three selected males showing the alternative sequence. Public reads from a Thai female and an Australian male supported the contrasting local structures. Longer reads crossed the gaps in other samples but did not connect the focal female’s chromosome copies. These findings identify specific sequence differences for future studies of sex determination in shrimp.

## 1. Introduction

Sex chromosomes can differ in sequence organization and inheritance despite having similar morphology [1,2]. In recently evolved or weakly differentiated sex chromosome systems, sex determination may be associated with a small chromosomal region, while the two sex chromosomes retain extensive sequence similarity across the remainder of their length [3]. Recombination may cease gradually, persist at low frequency, or change after turnover of the sex-determining locus [2,3]. Consequently, sex chromosomes may follow different evolutionary trajectories, characterized by varying degrees of sequence divergence and recombination suppression within and around the sex-determining region (SDR) [4]. Understanding these evolutionary relationships requires a detailed characterization of the genomic organization of the sex-associated region [4]. Statistical mapping identifies genomic regions associated with sex, whereas haplotype reconstruction reveals which variants are located on the same chromosome, how structural differences are organized, and how these differences relate to neighboring genes [5].

Studies of homomorphic sex chromosomes show why these distinctions matter. In *Aedes aegypti*, the strongly differentiated interval on the M-locus chromosome can be narrower than the region with little detectable recombination [6]. In guppies, rare recombination helps preserve Y-linked coding sequence, complicating a simple division between fully sex-linked and freely recombining regions [7]. Comparisons among cichlid populations reveal differences in the presence and organization of sex-linked blocks, raising questions about repeated recruitment and shared ancestry [8]. Together, these studies distinguish sex association, sequence differentiation, and recombination rather than treating their genomic boundaries as equivalent [6,7,8].

Long molecules provide haplotype information by linking sequence variants on the same chromosome copy [5,9]. Short reads provide limited long-range phase information, and repeats and heterozygous differences complicate assembly [5]. Long-read assemblies improve access to sex-limited and repeat-rich sequence. They also help identify collapsed homologous copies or switches between haplotypes [10,11]. Read-backed phasing links alleles observed on individual molecules, whereas haplotype-resolved assembly reconstructs longer sequence paths that can be checked against those reads [5,9]. Error-prone long reads can span intervals that shorter, more accurate reads leave disconnected; repeat-graph assembly can also recover local structure [12]. Combining these approaches helps separate physical coverage from allele identity within a common reference-coordinate framework.

Structural variants provide discrete features for comparing sex-linked regions. Insertions, inversions, and repeat expansions mark local structural differences within sex-linked regions [4,13]. They can also define candidates for testing regulatory effects on sex determination. Black carp provides one comparison, with a Y-specific, transposable-element-rich insertion near *dmrt1* [14]. Its regulatory context was investigated using haplotype-resolved genomes, population genotypes, allele-specific expression, and reporter assays. Silver arowana provides another, with a W-specific sequence near *foxl2* [15]. Analyses combined population association, chromatin contacts, developmental expression, and knockdown experiments. These studies connect structural reconstruction to tests of regulation and organismal effects. In a newly characterized SDR, reconstructing a local variant supplies a defined sequence for subsequent functional investigation.

Population sampling and species comparisons are needed to interpret local sequence architecture in an evolutionary context. Studies of knifejaw, seahorses, and halfbeak fish combined sex-specific or homologue-resolved assemblies with population genotypes, linkage disequilibrium, divergence estimates, phylogenetic comparisons, or structural-event dating [16,17,18]. Comparisons across geckos examined recurrent genomic predisposition across sex chromosome systems [19]. Horseshoe crab and starry flounder studies illustrate additional approaches to resolving sex-linked regions [20,21]. Closely related species need not show the same pattern. One stickleback comparison detected differentiation in *Pungitius pungitius*, but not in the sampled *P. sinensis* or *P. tymensis* [22]. A local haplotype map identifies sequence features and intervals for these broader tests, but cannot replace population or recombination evidence.

Crustaceans provide useful comparisons for studying differentiation between morphologically similar sex chromosomes. The Chinese mitten crab has morphologically similar putative Z and W chromosomes [23], whereas the black tiger shrimp has a highly repetitive genome [24]. Linkage and transcriptome-assisted maps support a ZW system in the Chinese mitten crab. *Artemia* provides a separate example involving W-chromosome evolution and unusual reproductive modes [23,25]. In black tiger shrimp, *Penaeus monodon*, linkage maps and sex-associated markers from several populations support female heterogamety, conventionally described as ZW females and ZZ males [26,27,28]. High-molecular-weight DNA methods and chromosome-level assemblies facilitate direct examination of the underlying sequence [24,29,30]. A recent SNP-array study also identified sex-associated variation in breeding material [31]. These resources localize sex-associated variation, but its arrangement into local female-associated haplotypes remains incompletely resolved.

Here, we investigated local W-like and Z-like haplotype architecture within the previously defined Chr19 candidate SDR of *P. monodon*. Our earlier association and marker study identified Chr19:2.71–2.89 Mb and addressed candidate-gene prioritization and marker validation. The present study used this fixed interval and the same family resources to examine local allele arrangements. We combined PacBio high-fidelity (HiFi) read-backed phasing from a marker-supported ZW individual with 311 exact sex-associated single-nucleotide polymorphisms (SNPs) and segregation in 200 full-sib offspring. We then examined how the phase blocks related to nearby genes and reconstructed INS00000105, a 54 bp insertion identified in the present structural-variant analysis. Public long reads addressed two separate questions: whether the local insertion-bearing structure recurred outside PMFXX, and whether longer molecules spanned the unresolved gaps. Thai female PacBio continuous long reads (CLR) provided an external structural comparison, Australian male CLR provided a documented male comparator, and Chinese Oxford Nanopore reads provided long-range physical evidence. This design links local orientation, gene context and structural reproducibility while retaining sample identity as the criterion for joining haplotypes. It provides a focused framework for investigating localized differentiation within the previously defined candidate SDR.

## 2. Materials and Methods

### 2.1. Study Design and Reference Genome

We examined local sequence differences within a previously mapped candidate SDR on chromosome 19. HiFi reads from one marker-supported ZW female (PMFXX) provided the focal haplotype reconstruction. Genotypes from one full-sib family, comprising 100 females and 100 males, assigned female-associated allele backgrounds to the reconstructed blocks. Gene annotation, local assembly, PCR/Sanger sequencing, and public long reads were used to examine specific genes and structural variants.

The PCR/Sanger follow-up used 20 female and 20 male market-sourced shrimp, as detailed in Section 2.5. These shrimp were separate from the discovery family and were part of the earlier 50-shrimp market panel. Animal procedures were approved by the institutional Animal Care and Use Committee (approval no. nhdf2025-49). All coordinates refer to PMFXX.genome.fasta and its corresponding GFF annotation. We fixed the candidate SDR at Chr19:2.71–2.89 Mb using the preceding association study. The wider interval, Chr19:2.6–3.1 Mb, was used only to display coverage and repeat context (Figures S1 and S4).

### 2.2. HiFi Variant Calling and Haplotype Assignment

Five validated Kompetitive allele-specific PCR (KASP) loci and two structural marker loci supported the focal female’s ZW classification. We aligned HiFi reads using minimap2 v2.26 with the HiFi preset [32]. We called variants across Chr19:2,713,000–3,026,000 using DeepVariant v1.10.0 with the PACBIO model [33], then normalized them using BCFtools v1.19 [34]. We retained PASS heterozygous SNPs and indels with depth ≥ 5 and genotype quality ≥ 20. WhatsHap v2.8 used the aligned reads to group variants into phase blocks [9].

We assigned local allele backgrounds using 311 fully called sex-associated SNPs. At these sites, all 100 female offspring were heterozygous, whereas all 100 males shared the same homozygous genotype. An informative SNP identified the female-associated allele within its phase block. We labeled the corresponding background W-like and the alternative background Z-like only when all informative SNPs agreed. These labels describe family-associated alleles, not demonstrated chromosome-wide or universally sex-specific sequences. We retained the original HP1/HP2 haplotype labels because their numbering is assigned independently within each block. When testing connections between blocks, we grouped alignments originating from the same read.

### 2.3. Segregation Within the Discovery Family

We matched phased sites to the family SNP/indel variant call format (VCF) files by position and alleles. Fully called diploid genotypes were retained without additional per-genotype depth or quality filtering; we recorded missingness and quality. For each shrimp, the W-like carrier fraction was the proportion of called sites carrying at least one W-like allele. This score covered the complete matched block, including the portion of PS2835647 beyond 2.89 Mb. The matched sites numbered 391/552 in PS2729206 and 147/199 in PS2835647. We treated a genotype as missing if either allele was absent.

We screened core sex-associated markers using the following criteria: missingness ≤ 10% in each sex, available sex-specific mean depth ≥ 5, and Benjamini–Hochberg q < 0.05. Additional criteria were female carrier fraction ≥ 0.90, male carrier fraction ≤ 0.05, and genotype–sex concordance ≥ 0.90. Multiple-testing correction included finite per-marker Fisher-test *p* values across both oriented blocks. We compared W-like carrier fractions using two-sided Mann–Whitney tests. We compared INS54 carrier counts using a two-sided Fisher exact test, excluding uncalled genotypes. These analyses describe segregation within the discovery family, not independent tests of population-wide sex specificity.

### 2.4. Gene Overlap and Sequence Comparison

We measured overlap between phase blocks and the gene bodies, coding sequences (CDS), and strand-aware 3 kb upstream regions of genes23–27. Near-complete local reconstruction required both haplotypes to cover ≥85% of the gene body, ≥95% of the CDS, and ≥90% of splice regions.

We reconstructed gene24 haplotype sequences by applying phased variants to the reference gene model. We compared protein sequences using a custom Smith–Waterman local-alignment function implemented in Python 3.11.9 with NumPy 1.26.4. Scores were +2 for a match, −1 for a mismatch, and −2 for a linear gap penalty. Amino acid identity was the number of identical non-gap positions divided by the complete gapped alignment length. The function and reference-protein comparison are provided in Supplementary Method S1.

### 2.5. INS54 Reconstruction and PCR/Sanger Sequencing

We inspected HiFi reads spanning the insertion junction at Chr19:2,741,735|2,741,736. We assembled HP1 and HP2 reads separately using hifiasm v0.25.0-r726 and Flye v2.9.6-b1802 [5,12], then aligned the assemblies using minimap2 v2.26-r1175. Structural support required agreement between the reads and a haplotype-specific contig spanning both insertion junctions.

We performed flanking PCR in 20 female and 20 male market-sourced shrimp. These shrimp were separate from the discovery family, but were part of the preceding study’s 50-shrimp panel. Primers were A-F (5′-GAATCCTCCGTGATTTGCTTGG-3′) and A-R (5′-GAGCACCGTTCTATTTAGTTTACGT-3′). We used 2×TransStart FastPfu PCR SuperMix (TransGen Biotech, Guangzhou, China), with 33 cycles, 53 °C annealing, and 1% agarose gels. HiFi reads containing both exact primer sites predicted amplicons of 832–843 bp for HP1 and 1031–1043 bp for HP2. This length difference reflects the complete intervening sequence, not INS54 alone. The uncropped gel is provided in Figure S5.

We selected three females (0–22, 0–24, and 0–25) and three males (1–22, 1–24, and 1–25) for Sanger sequencing. Each shrimp was amplified in three 50 µL reactions. PCR products were sent to Tsingke for band excision and bidirectional sequencing.

### 2.6. Public Long-Read Comparisons at INS54

We checked public PacBio CLR/subreads against their archive accessions and source publications. The Thai dataset contained 39 runs from PRJNA611030/SAMN14330694; the publication describes a 5-month-old female muscle donor [24]. The Australian dataset contained 34 runs from PRJNA590309/SAMN13324362, a documented male muscle donor [30]. We aligned the runs to the same PMFXX reference using minimap2 -x map-pb and retained run-specific read groups.

We grouped Thai subreads by native zero-mode waveguide (ZMW) identifiers to avoid repeatedly counting one molecule. We used Australian archive read identifiers as proxies where native ZMW identity was unavailable. Structural calls required either the insertion sequence with its flanks or a continuous no-insertion junction. Assignment of local allele backgrounds in PS2729206 required ≥3 informative sites and ≥80% agreement. A stricter check required ≥5 sites and ≥90% agreement. A separate comparison against symmetric insertion/no-insertion templates is shown in Figure S3.

### 2.7. Long-Read Spans Across the Phase Gaps

For Gap1 (Chr19:2,743,494–2,787,163), we required a single Thai ZMW to reach phased positions on both sides in its primary alignment. We inspected clipping, supplementary alignments, mapping quality, and local sequence. Physical span and local allele assignment were evaluated separately.

We screened six Oxford Nanopore (ONT) runs from PRJNA1365493 (China) for reads spanning Gap2. Candidate reads were mapped competitively to Chr19:2.68–2.92 Mb and 44 non-target reference segments, termed decoys. Decoys were selected from reference similarity hits with length ≥ 500 bp and identity ≥ 80%, with 2 kb flanks. Table S1 lists their coordinates, selection criteria, identities, and alignment lengths. A gap-spanning read required ≥500 aligned bases on each side, mapping quality (MAPQ) ≥ 30, and alignment edit distance (NM)/aligned length ≤ 0.15. We excluded reads with a deletion or skipped region ≥ 1000 bp, or a decoy alignment score ≥90% of the target score.

## 3. Results

### 3.1. Three Phase Blocks Intersect the Chr19 Candidate SDR

**Figure 1:**
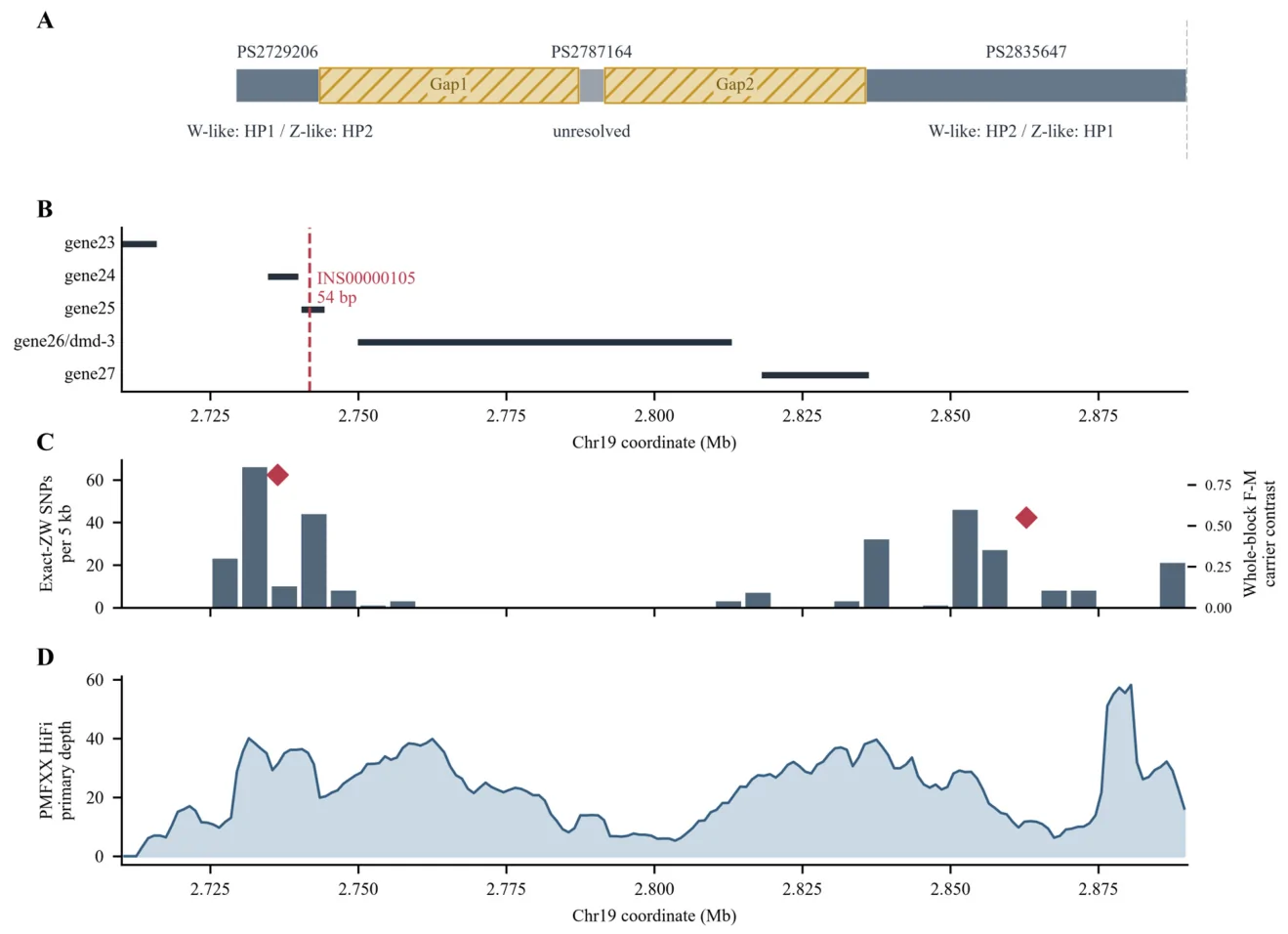
Phase blocks and gene context on chromosome 19. (**A**) Block locations and unresolved gaps (hatched amber). Neutral block colors do not assign W/Z identity; the original HP1/HP2 haplotype labels are retained. PS2835647 extends beyond the 2.89 Mb SDR boundary to 2.917304 Mb. (**B**) Gene models and INS54. (**C**) Sex-associated SNP density and median female–male W-like carrier-fraction differences (red diamonds) over whole blocks, including sequence outside the displayed interval. (**D**) Primary HiFi read depth for the focal female.

We first asked which Chr19 variants were linked within the same individual. HiFi read-backed phasing grouped linked heterozygous variants, and three resulting blocks overlapped the candidate SDR (Figure 1). Each block contains variants connected by the focal female’s reads. The intervening gaps therefore mark unresolved allele connections, rather than established breaks in the chromosome.

Across the variant-calling interval, 1099 heterozygous variants, comprising 996 SNPs and 103 indels, formed four phase blocks. Three blocks overlapped the candidate SDR. These were PS2729206 (Chr19:2,729,206–2,743,493; 552 variants), PS2787164 (2,787,164–2,791,655; 12 variants), and PS2835647 (2,835,647–2,917,304; 199 variants). The fourth block contained 336 variants and lay outside the candidate SDR.

### 3.2. Family Markers Assign Female-Associated Alleles in Two Blocks

**Figure 2:**
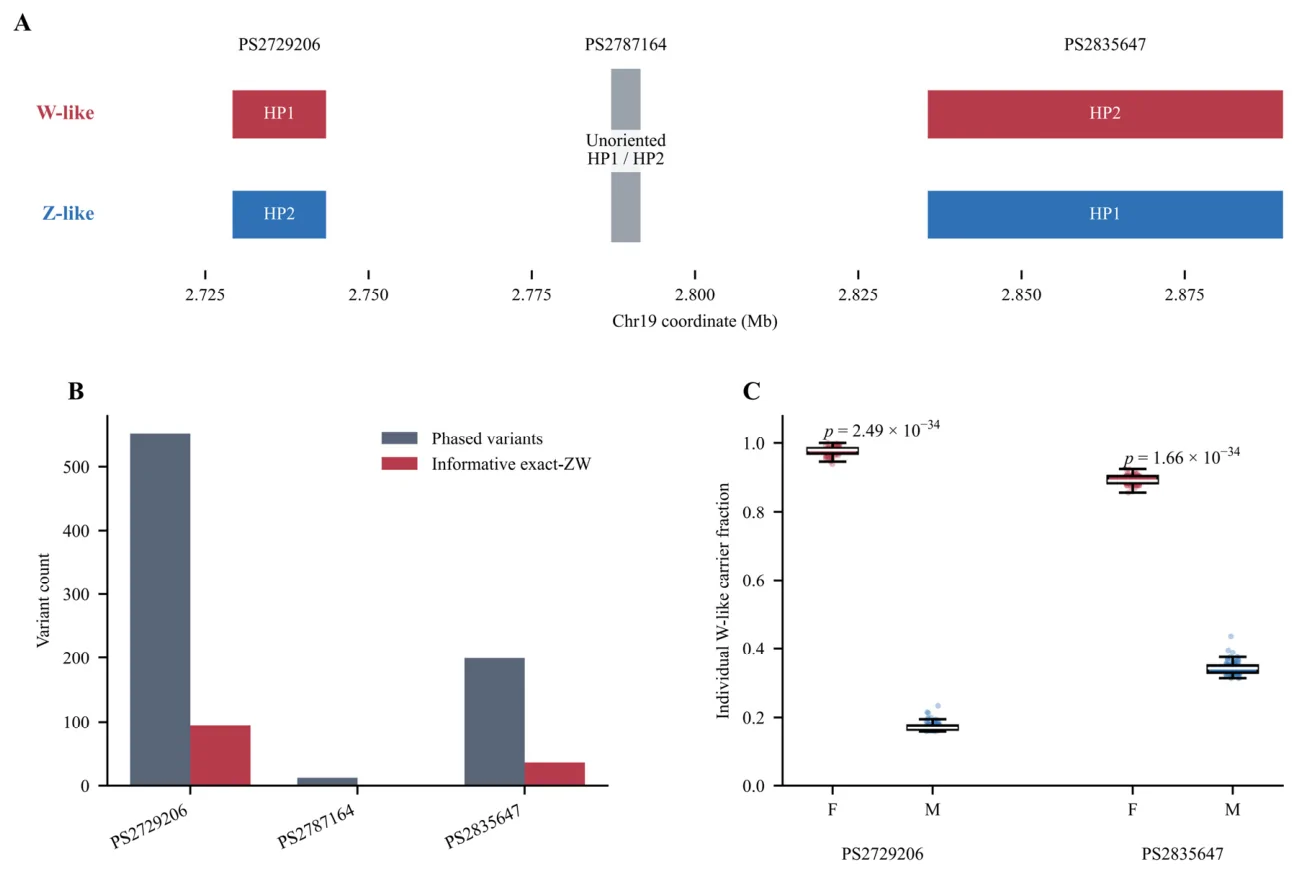
Local haplotype assignments and segregation in the discovery family. (**A**) Female-associated (W-like, red) and alternative (Z-like, blue) allele backgrounds; the central block is unoriented (gray). HP labels are specific to each block. (**B**) Numbers of phased variants and informative sex-associated SNPs. (**C**) W-like carrier fractions in 100 females and 100 males across the complete matched blocks. Boxes show medians and interquartile ranges; whiskers use the 1.5× interquartile-range rule. Nominal Mann–Whitney *p* values describe within-family differences, not independent validation.

We next asked whether the local haplotypes could be assigned to female-associated and alternative sequence backgrounds. We mapped the 311 sex-associated family SNPs to the phase blocks and examined their allele assignments. In PS2729206, 95 SNPs assigned HP1 as W-like and HP2 as Z-like. In PS2835647, 36 SNPs assigned HP2 as W-like and HP1 as Z-like. All 131 informative SNPs agreed within their respective blocks (Figure 2A). Because haplotype labels are assigned independently within each block, HP1 does not identify the same sex-associated background throughout the interval. Figure 2 places each W-like background above its Z-like counterpart while retaining the original HP labels.

The central block contained no informative sex-associated SNP that could assign its allele background. The map therefore identifies two oriented local allele pairs and a separately phased, unoriented central block. Their order is known in reference coordinates, but their connections within the focal female remain unresolved.

The two oriented blocks retained 293 and 90 core sex-associated markers, respectively. Across all called matched sites, median W-like carrier fractions in PS2729206 were 0.980 in females and 0.169 in males (*p* = 2.49 × 10^−34^). Corresponding medians in PS2835647 were 0.891 and 0.341 (*p* = 1.66 × 10^−34^; Figure 2C).

The female–male median differences were 0.810 for PS2729206 and 0.550 for PS2835647. These summaries relate the reconstructed sequences to sex-associated inheritance within the family. They measure allele carriage, not nucleotide divergence or chromosome-wide W specificity.

### 3.3. The Phase Map Distinguishes Gene24 from Intact dmd-3

**Figure 3:**
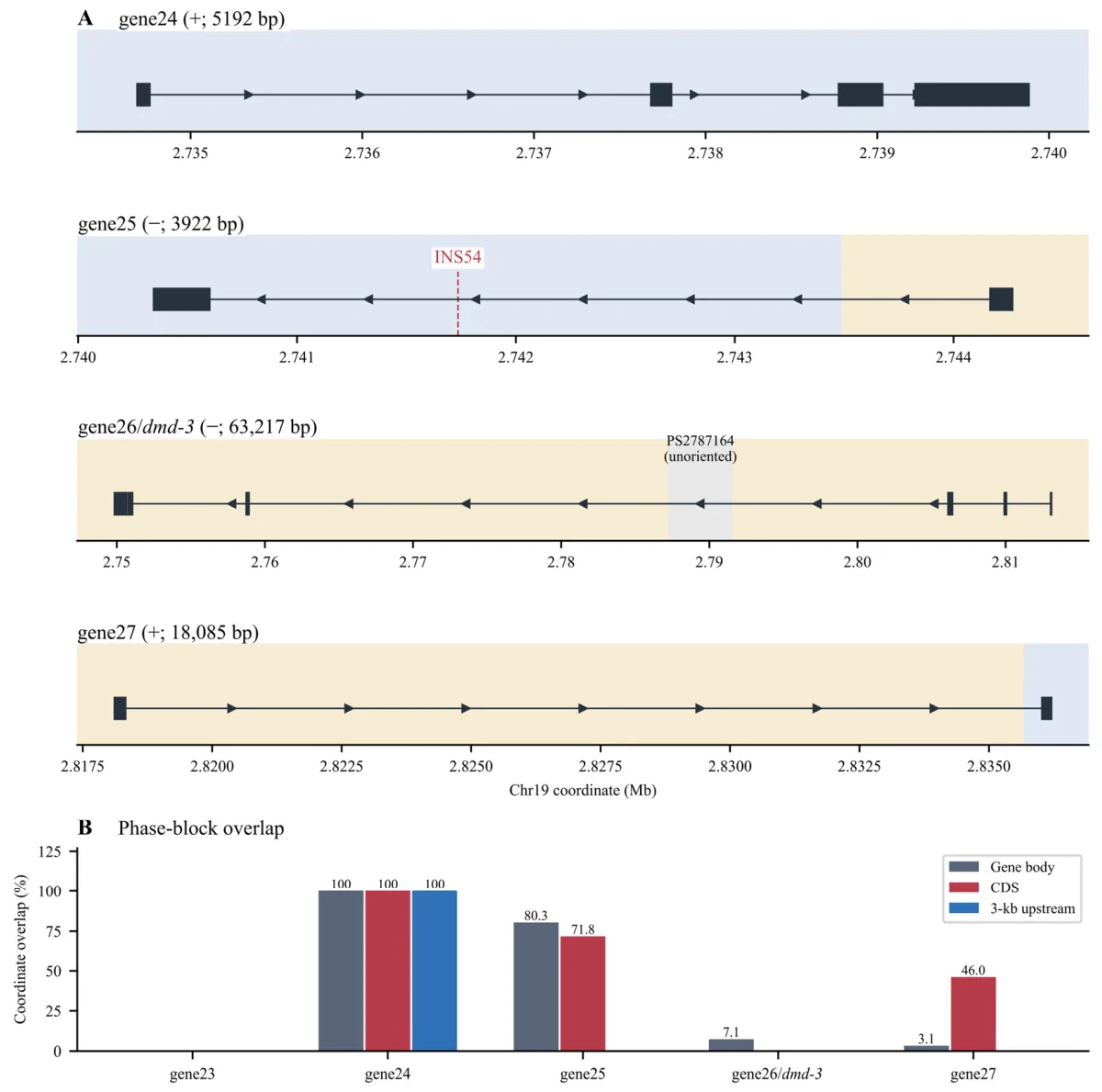
Gene overlap with the phase blocks. (**A**) Gene24–27 models, each shown on its own coordinate scale. Boxes indicate coding sequences (CDS), lines indicate introns, and arrows indicate strands. Blue-gray shading marks oriented blocks, gray marks the unoriented central block, and pale amber marks unresolved gaps. INS54 lies in gene25 intron 1. (**B**) Percentages of the complete gene body, CDS, and strand-aware 3 kb upstream region that overlap any phase block. Gene23 includes sequence outside the candidate SDR. Gene24 lies entirely within an oriented block, whereas the 7.11% gene-body overlap for gene26/*dmd-3* is with the unoriented central block. These fractions measure coordinate overlap, not the completeness of independently assembled sequences.

We examined which genes in the candidate SDR could be compared as local W-like and Z-like allele sequences. We mapped annotated genes to the phase blocks and compared sequences within the oriented regions. Gene24 lay within the first oriented block, permitting comparison of its reference-anchored W-like and Z-like sequences. These sequences differed at 181 SNP and 14 indel positions and had 94.88% identity. Gene25 extended from this block into Gap1 (Figure 3).

Protein comparisons distinguished the local gene24 model from the intact gene26/*dmd-3* locus. Gene24 matched the C-terminal region of gene26/*dmd-3* over a 308-aa alignment with 87.34% identity. Because its annotated model lacks the DM DNA-binding domain, we describe gene24 as a partial *dmd-3*-related model. The intact gene26/*dmd-3* locus spans 63,217 bp across Gap1, the central block, and Gap2. Only 7.11% of its gene body overlapped the unoriented central block. Its six CDS exons lay outside confidently oriented blocks, leaving its W-like and Z-like coding sequences unresolved. Gene27 crossed Gap2 and the third block; 3.07% of its body and 46.03% of its CDS overlapped that block (Figure 3).

### 3.4. INS54 in gene25 Intron 1 Defines a Local W-like Structural Difference

**Figure 4:**
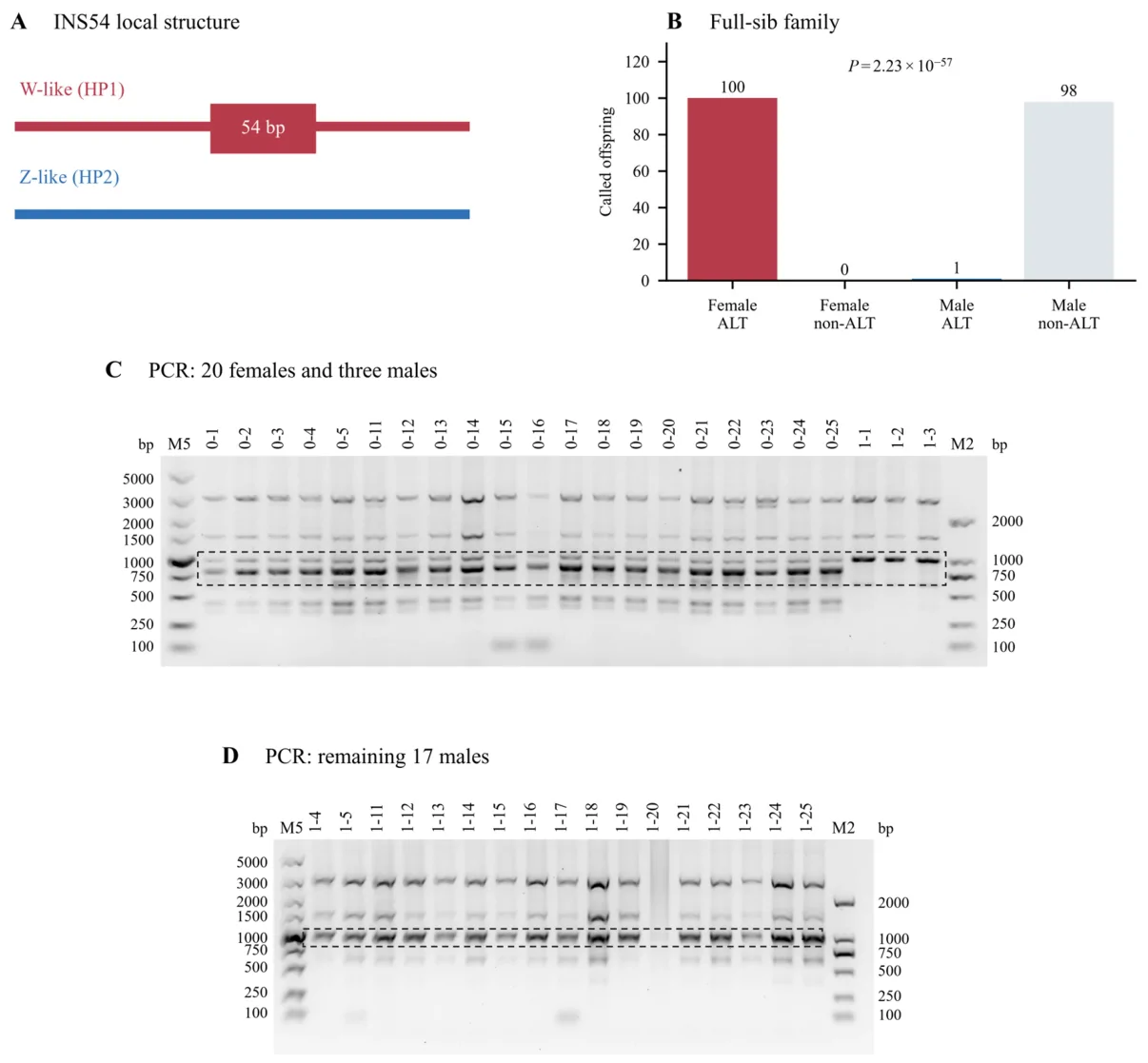
INS54 structure, family segregation, and flanking PCR. (**A**) Insertion-bearing W-like HP1 and no-insertion Z-like HP2 configurations, supported by 17 HiFi reads and two local assemblers. The schematic is not to scale. (**B**) Carrier counts among called offspring; one male was uncalled. (**C**,**D**) Contiguous upper and lower gel rows. C contains females 0–1–0–5 and 0–11–0–25, followed by males 1–1–1–3. D contains males 1–4, 1–5, and 1–11–1–25. Prefixes 0/1 denote female/male samples. All 40 sample lanes are retained in their original order, without local intensity changes. M5/M2 denote GL DNA Marker 5000/Trans2K DNA Marker; ladder sizes are in bp. Dashed boxes mark the two-band region in (**C**) and the principal male-band region near 1.04 kb in (**D**). Band positions alone do not establish INS54 genotypes. The shrimp are a subset of the earlier 50-shrimp market panel. Figure S5 shows the uncropped source gel.

We examined INS00000105 (INS54) in gene25 intron 1 as a structural feature of the W-like allele background. We compared its support from reads, haplotype-separated local assemblies, family genotypes, and targeted PCR/Sanger sequences. INS54 was a 54 bp insertion between Chr19 positions 2,741,735 and 2,741,736 within the first oriented block. All 17 supporting haplotagged HiFi reads belonged to W-like HP1. Thirteen carried 54 inserted bases, three carried 53, and one carried 55 (Figure S2). Both hifiasm and Flye assemblies spanned the insertion junctions. Their Z-like assemblies supported the alternative no-insertion structure (Figure 4A).

Family genotypes linked the reconstructed insertion to sex-associated segregation. INS54 was called in all 100 females and 1/99 called males; one male was uncalled (Fisher exact *p* = 2.23 × 10^−57^; Figure 4B). The insertion lies in gene25 intron 1, 2532 bp from the transcription start site and 1135 bp from the nearest annotated splice boundary. It was outside annotated repeats and 145 bp from the nearest simple repeat.

We performed flanking PCR in 40 market-sourced shrimp to examine local sequence states outside the discovery family. Band patterns differed between the sexes (Figure 4C,D). Females 0–23, 0–24, and 0–25 had a prominent band near 0.84 kb and a weaker band near 1.04 kb. Males 1–1, 1–2, and 1–3 had a prominent band near 1.04 kb.

**Figure 5:**
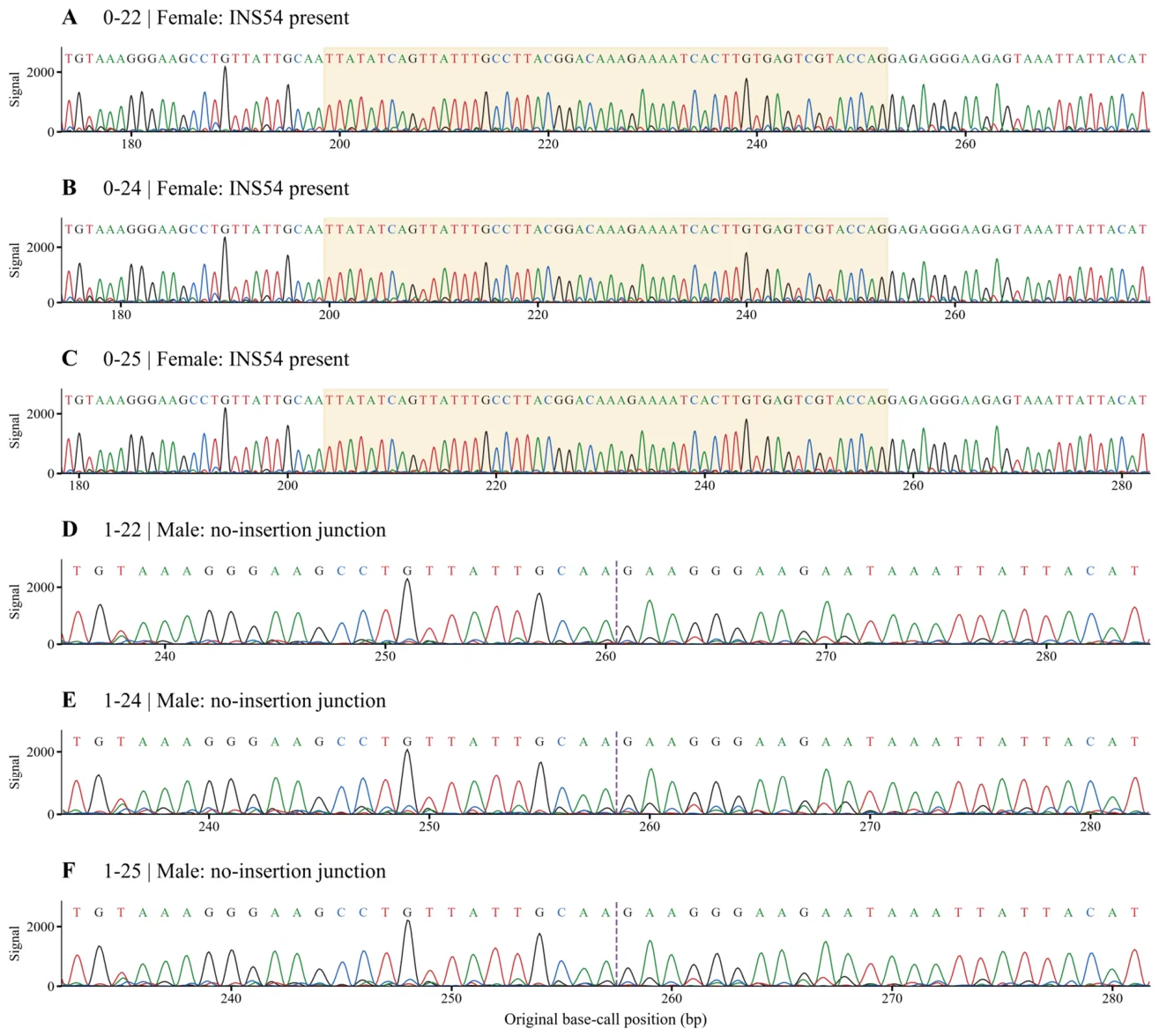
Local Sanger sequences from six selected shrimp. (**A**–**C**) Females 0–22, 0–24, and 0–25. INS54 is shaded, and both 25 nt flanks are shown. (**D**–**F**) Males 1–22, 1–24, and 1–25. The dashed line marks the no-insertion junction. Panels represent six biological individuals, not population frequencies or homozygosity calls. Trace and base-call colors identify A (green), C (blue), G (black), and T (red).

Sanger reads from the selected shrimp directly supported the two local sequence configurations. Forward reads from females 0–22, 0–24, and 0–25 contained INS54 and 25 nt on each side (Figure 5A–C). Each provided a contiguous 104 nt match to the PMFXX W-like sequence. All insertion bases had Phred Q ≥30, with minimum values of 36, 30, and 52, respectively. Predominant forward sequences from males 1–22, 1–24, and 1–25 crossed the no-insertion junction (Figure 5D–F). These six shrimp support contrasting local structures, but do not establish genotype frequencies in the complete market panel.

### 3.5. Long Molecules Extend Physical Coverage Across the Two Phase Gaps

**Figure 6:**
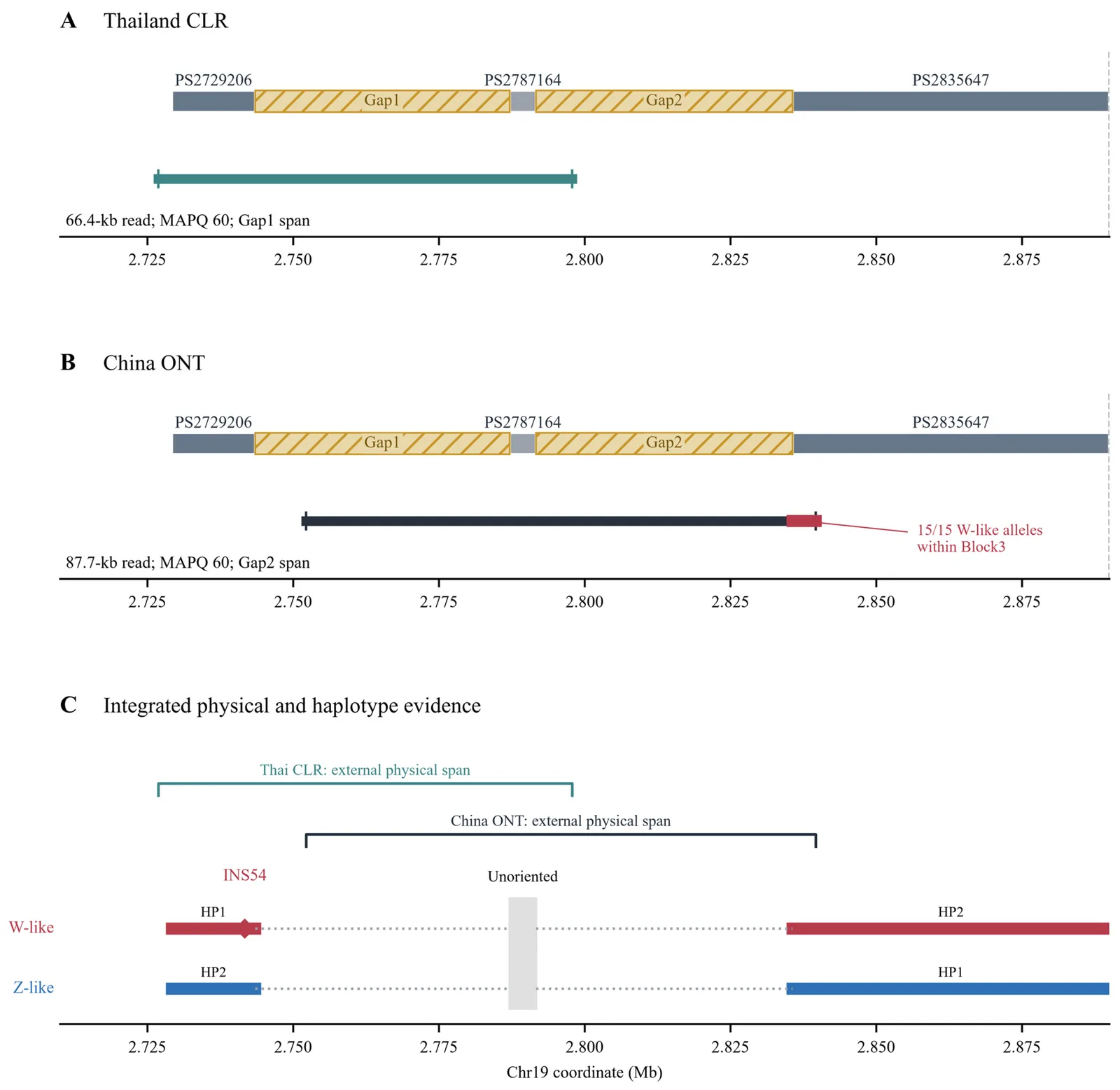
External long-read spans and unresolved focal haplotypes. All panels use PMFXX coordinates. (**A**) One 66.4 kb Thai PacBio continuous long read (CLR) from a single zero-mode waveguide (ZMW) from SRR12622224 (SAMN14330694) spans Gap1. (**B**) One 87.7 kb Chinese Oxford Nanopore (ONT) molecule, SRR36327749.149556 (SAMN53693758), spans Gap2. Both alignments have mapping quality (MAPQ) 60. The red terminal segment in (**B**) marks overlap with PS2835647 and its 15 W-like informative alleles, not individual SNP positions. The Thai publication reports a female donor; the Chinese donor’s sex and identity relative to PMFXX are unconfirmed. (**C**) Separately assigned focal W-like/Z-like blocks, INS54, and the unoriented central block. Dotted lines mark unresolved PMFXX phase connections. Brackets indicate external physical spans, not connections between PMFXX chromosome copies.

We screened public Thai CLR and Chinese ONT reads to determine whether continuous molecules could span the intervals between local phase blocks. Physical spans and local allele backgrounds were evaluated separately. A 66,430 bp Thai CLR subread from SRR12622224 spanned Gap1. Its primary alignment extended from Chr19:2,726,889 to 2,797,845 with MAPQ 60 and reached phased sequence on both sides (Figure 6A). After grouping subreads, the observation represented one ZMW from SAMN14330694. It established a physical path across Gap1, but its local allele background remained unassigned.

A Chinese ONT molecule provided a corresponding physical span across Gap2. Read SRR36327749.149556 was 87,707 bp long and aligned from Chr19:2,752,229 to 2,839,636. It had MAPQ 60 and an NM fraction of 0.02029, and met the target/decoy criteria (Figure 6B). Its terminal overlap with PS2835647 carried 15/15 PMFXX-defined W-like informative alleles. Removing an identical archive record left one molecule from SAMN53693758. This molecule combined a physical span across Gap2 with local W-like allele context at its Block3 end.

The two external molecules provided physical paths across the gaps in their respective donors. They extended coverage beyond the focal phase blocks and identified intervals for further haplotype reconstruction (Figure 6C). These observations remained separate from the unresolved phase connections between the focal female’s blocks.

### 3.6. External CLR Supports Reciprocal Local Structures and Allele Context

**Figure 7:**
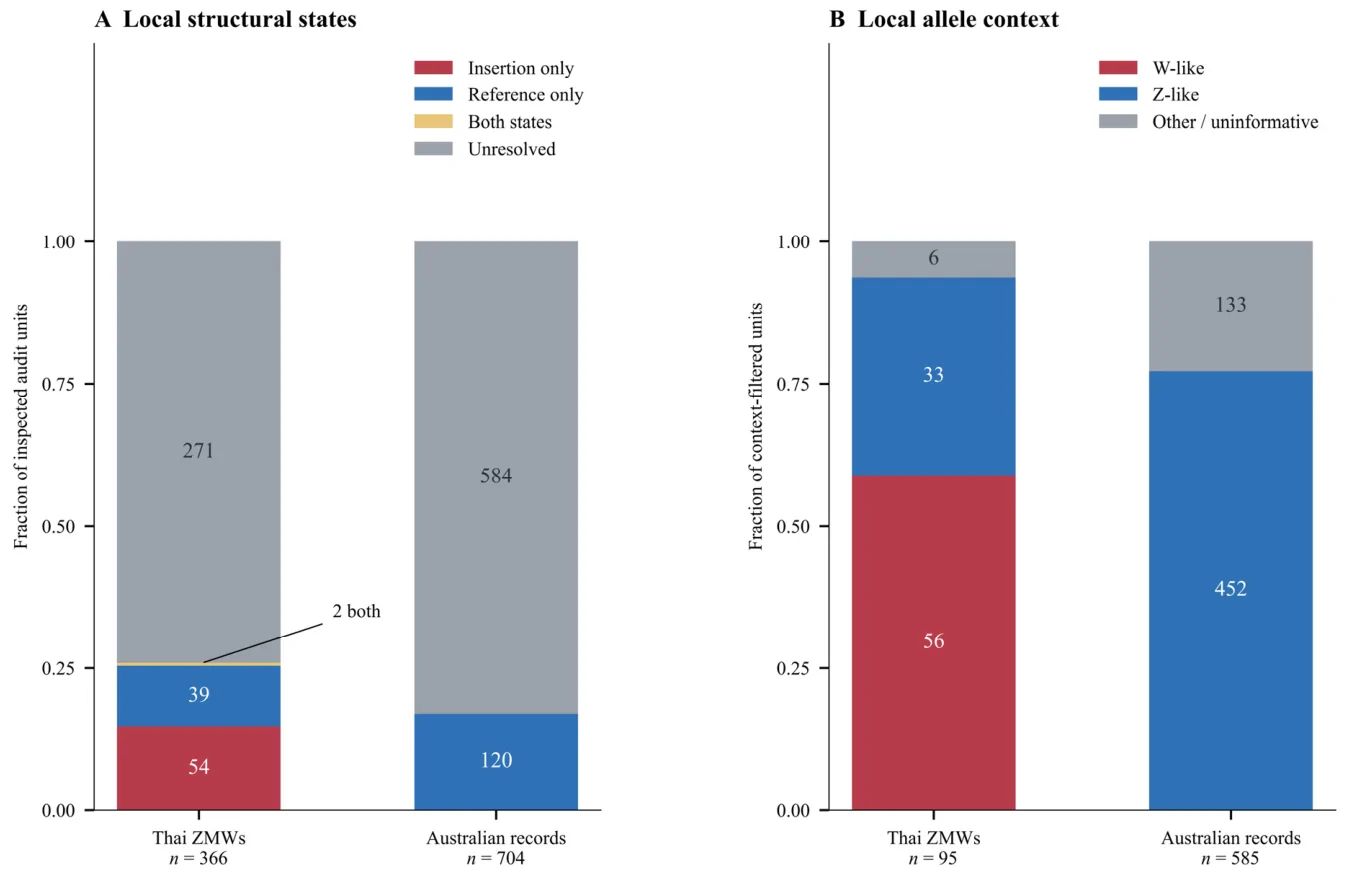
Structural calls and local allele backgrounds in public CLR datasets. (**A**) Insertion-only/no-insertion-only/both/unresolved counts are 54/39/2/271 for Thailand (366 ZMWs) and 0/120/0/584 for Australia (704 archive records). (**B**) Local context calls from 95 Thai ZMWs and 585 Australian records. Thai counts are 56 W-like, 33 Z-like, and 6 uninformative calls. The 87-ZMW test in the text excludes the six uninformative and two dual-state W-like molecules. Panels use different eligibility criteria. Native ZMW independence is unverified for the Australian record proxies. Each dataset represents one BioSample, a publication-reported Thai female or a documented Australian male. These read-level counts are not population allele frequencies. Figure S3 presents the separate symmetric-template comparison.

We next asked whether the gene25 insertion-bearing structure recurred outside PMFXX and retained the same local sex-associated allele background. We compared INS54 structural states and linked informative alleles in Thai and Australian CLR datasets. The Thai dataset contained both structural states. Among 366 inspected ZMWs, 54 supported insertion only, 39 supported no insertion only, 2 supported both states, and 271 supported neither resolved state (Figure 7A). A separate allele-context screen retained 95 ZMWs, with 56 W-like, 33 Z-like, and 6 uninformative calls (Figure 7B). The structure–context test included 87 eligible molecules. Insertion-only calls comprised 52 W-like and 0 Z-like ZMWs; no-insertion calls comprised 2 W-like and 33 Z-like ZMWs (Fisher exact *p* = 6.64 × 10^−17^). INS54 was therefore associated with PMFXX-defined local W-like alleles within this Thai sample.

The Australian male CLR dataset from SAMN13324362 [30] provided a comparison with the no-insertion state (Figure 7A,B). Among 704 inspected records, 120 supported no insertion, none supported insertion, and 584 were unresolved. Of 585 context-filtered records, 452 met the standard Z-like rule, and none met the W-like rule. The separate symmetric-template comparison supported the same structural contrast (Figure S3). Insertion/no-insertion/unresolved counts were 17/25/5 for PMFXX HiFi, 61/31/26 for Thai CLR, and 0/71/159 for Australian CLR. These analyses supported the local insertion-bearing and no-insertion structures under their respective selection criteria.

## 4. Discussion

### 4.1. From a Sex-Associated Interval to Local Haplotype Architecture

Our analysis resolved local allele arrangements within a previously mapped sex-associated interval, while leaving connections between phase blocks unresolved. PS2729206 contained the insertion-bearing W-like state and the complete annotated gene24 model. A second oriented block provided a separate female-associated sequence anchor. The central block remained unoriented, showing that the available phase information was uneven across Chr19:2.71–2.89 Mb. Gene24 could be compared within an oriented block, whereas intact gene26/*dmd-3* crossed both gaps and the unoriented central block. The map therefore distinguishes local allelic comparisons from gene sequences that still require reconstruction.

### 4.2. Family Segregation Connects Local Haplotypes to Sex-Associated Inheritance

Family segregation associated the reconstructed local haplotypes with female-associated inheritance. This observation complements earlier *P. monodon* linkage studies based on female-parent transmission of sex-linked markers [26]. The observed female–male median carrier contrast was larger for PS2729206 than for PS2835647. Scores across all called phased sites retained alleles also observed in males, describing broader backgrounds than perfectly sex-associated markers alone. The contrast between the blocks suggests that female association was not uniform across the sampled region.

These carrier fractions describe segregation within the discovery family, rather than an independent population test. They do not measure nucleotide divergence or establish a statistical difference between the effects of the two blocks. The PS2835647 summary also includes sequence beyond the displayed candidate-SDR boundary. The implications of reusing the family and market panels are considered in Section 4.6.

### 4.3. Public Long Reads Extend Structural Evidence Beyond the Focal Dataset

Public long reads extended physical coverage beyond the locally phased PMFXX sequences. Thai female CLR reproduced the insertion-bearing structure and spanned Gap1; Australian male CLR provided a no-insertion, predominantly Z-like local comparison. A separate, deduplicated Chinese ONT molecule spanned Gap2 and carried W-like alleles within its Block3 overlap. These complementary observations illustrate the value of combining accurate allele assignments with longer physical spans when examining sex-linked sequence [10,11]. They extend the structural comparisons beyond PMFXX, but do not join its phase blocks.

A span in an external donor does not establish structural identity or allelic continuity with PMFXX. The observations therefore support donor-specific physical paths and recurrence of local structures, rather than a continuous focal haplotype. Connecting PMFXX blocks still requires phase-informative molecules from that individual or independently established sample identity.

### 4.4. INS54 and the Gene Map Provide Specific Functional Targets

INS00000105 provides a defined structural feature for investigating the family-associated W-like sequence. HiFi molecules and concordant local assemblies support both insertion junctions, while Thai and Australian CLR support contrasting local structures (Figure 7A,B). Selected female Sanger reads contain INS54 and its flanks; predominant sequences from the selected males cross the no-insertion junction. This local contrast does not assign every gel-band class or genotype in the market panel.

The location of INS54 in gene25 intron 1 makes transcript usage and local regulation relevant questions for further study. Its distance from the nearest annotated splice boundary does not justify assuming direct disruption of a canonical splice site. The defined sequence difference nevertheless permits tests of insertion-associated splicing or expression.

The gene map also separates the partial *dmd-3*-related gene24 model from intact gene26/*dmd-3*. Although gene24 shares C-terminal sequence similarity with gene26, it lacks the DM DNA-binding domain and should not be treated as an equivalent intact transcription factor. Its local W-like and Z-like sequences permit copy-specific investigation. In contrast, gene26 coding haplotypes require further reconstruction before allelic functional comparisons can be made.

The black carp and silver arowana studies combined structural candidates with population genotypes, developmental expression, and regulatory or perturbation assays [14,15]. Those analyses extend beyond the sequence comparisons established here. In shrimp, full-length transcripts and copy-specific validation should first clarify the gene24 annotation and its relationship to gene26.

### 4.5. Implications for Investigating Sex Chromosome Differentiation

Earlier linkage and association studies support female heterogamety in *P. monodon* [26,27,28,31]. Within that framework, the sampled haplotypes support localized W-like sequence differentiation. Our map relates local variants to two oriented blocks and nearby genes, defining structural targets despite unresolved phase connections. Population and homologue-resolved studies in knifejaw, seahorses, and halfbeak fish illustrate approaches to testing sex chromosome history [16,17,18]. Stickleback and reptile comparisons also show why such interpretations require evidence specific to the lineage being studied [22,35].

### 4.6. Study Limitations and Future Directions

Several features of the study limit the scope of its conclusions. Marker screening, haplotype orientation, and segregation summaries reused the discovery family. Selection for sex concordance can enrich family-specific linked alleles, so agreement does not independently establish population-wide sex specificity. The market panel reused 40 shrimp from the preceding 50-shrimp panel, and Sanger sequencing examined only six selected shrimp. Unrelated families and geographically independent, sexed shrimp are needed to distinguish recurring sex-associated structures from private linked haplotypes.

The external long-read datasets also have important sampling limits. Each CLR dataset represents one donor, and sex and geography are confounded in the Thai–Australian comparison. Reference-based candidate selection and the 44-decoy screen restrict the alternative placements examined. Population-specific structural polymorphism therefore remains a possible explanation for differences between donors.

Phase-block boundaries reflect informative variants and available molecular links, not necessarily evolutionary strata or recombination boundaries. The unoriented central block records missing orientation information, rather than demonstrated similarity or differentiation between chromosome copies. Matched haplotype-resolved samples, population genotypes, and linkage analyses could test how broadly the local structural pattern is maintained and how it arose. The present phase gaps do not establish evolutionary age or recombination suppression.

## 5. Conclusions

We resolved local W-like and Z-like allele arrangements within the Chr19 candidate SDR in *P. monodon*. HiFi phasing and family markers oriented two allele pairs within three separate phase blocks. Gene24 could be compared locally, whereas the coding haplotypes of intact gene26/*dmd-3* remained unresolved. INS00000105, a 54 bp insertion in gene25 intron 1, provided a structural feature supported by HiFi reads, local assemblies, and family segregation. Selected Sanger sequences and external CLR comparisons supported contrasting local sequence states. Longer external molecules spanned the reference-coordinate gaps in their respective donors, but did not connect the PMFXX phase blocks. These findings identify local allele arrangements, an intronic structural difference, and gene-level targets for further reconstruction and functional investigation within the candidate SDR.

## Data Availability

The accession identifiers associated with this study are PRJNA1365493/SAMN53385223/SRR36145705 for PMFXX PacBio HiFi; PRJNA1365493 for the six ONT runs SRR36286560, SRR36305443, SRR36321516, SRR36327749, SRR36329896 and SRR36329897; PRJNA611030/SAMN14330694 for Thai PacBio CLR; and PRJNA590309/SAMN13324362 for Australian PacBio CLR. The supplied accession for the 200-offspring family resequencing data is PRJNA1515402.

## Supplementary Materials

The following supporting information can be downloaded at: https://www.mdpi.com/article/10.3390/biology15211962/s1. Figures S1–S4 provide coverage, repeat context and read-level sensitivity analyses. Figure S5 contains the uncropped PCR gel used in Figure 4C,D. Table S1 lists the primary ONT target and all 44 decoy sequences. Method S1 provides the protein-alignment algorithm and its reference-sequence result.
