# Supplementary material for: Local W-like Haplotype Architecture in the Candidate Sex-Determining Region of Black Tiger Shrimp
Source: Biology (Basel). 2026 Nov 1;15(21):1962. doi: 10.3390/biology15211962 (PMC13761503; doi:10.3390/biology15211962)
Supplement: Supplementary file 1 [file biology-15-01962-s001.zip › biology-4599739-supplementary.pdf]

Figures S1–S5, Table S1 and Supplementary Method S1

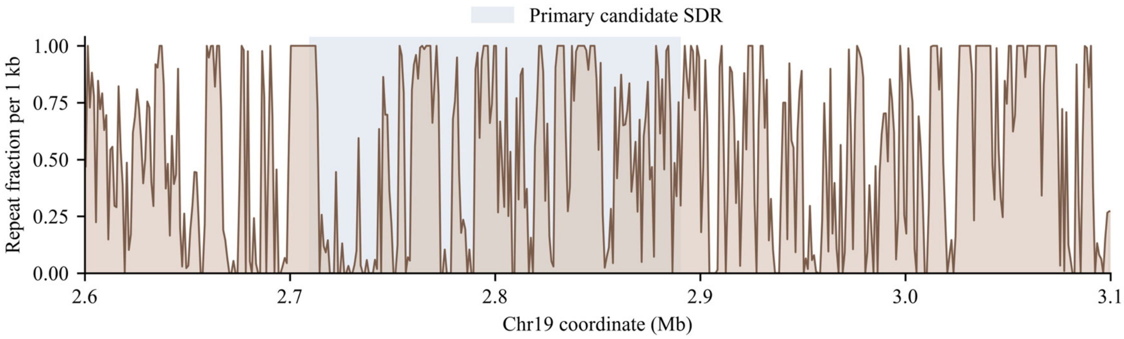

Figure S1. Repeat-sequence landscape across Chr19:2.6–3.1 Mb. Repeat-annotated fractions are shown for all 500 original 1-kb bins. Shading marks the primary candidate SDR, Chr19:2.71–2.89 Mb.

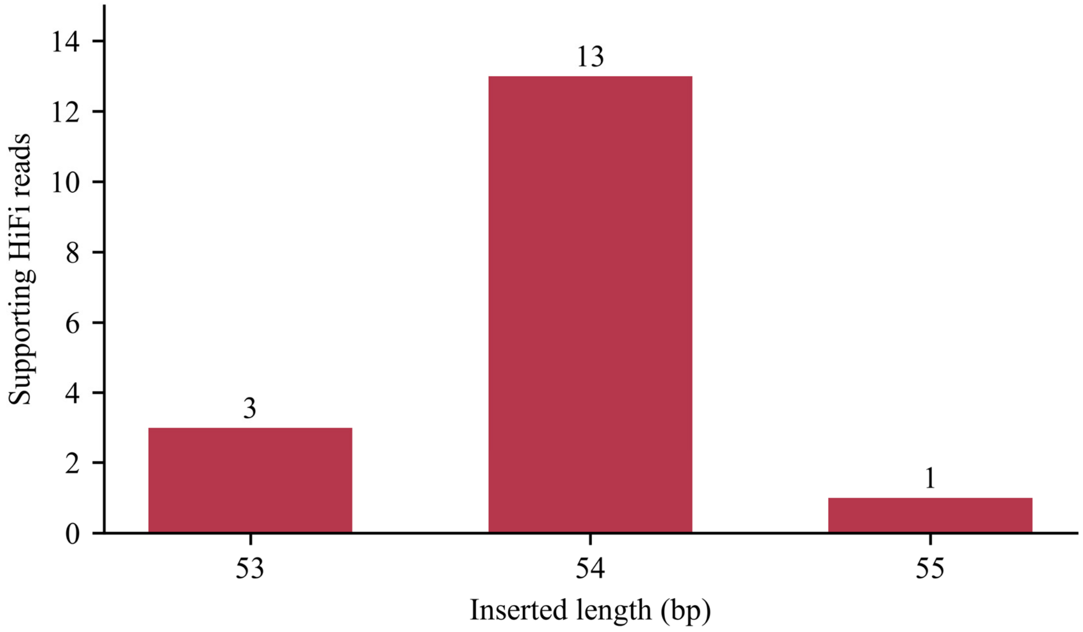

Figure S2. Inserted-length distribution among 17 W-like HiFi reads. There are 3 reads with 53 bp, 13 with 54 bp and 1 with 55 bp.

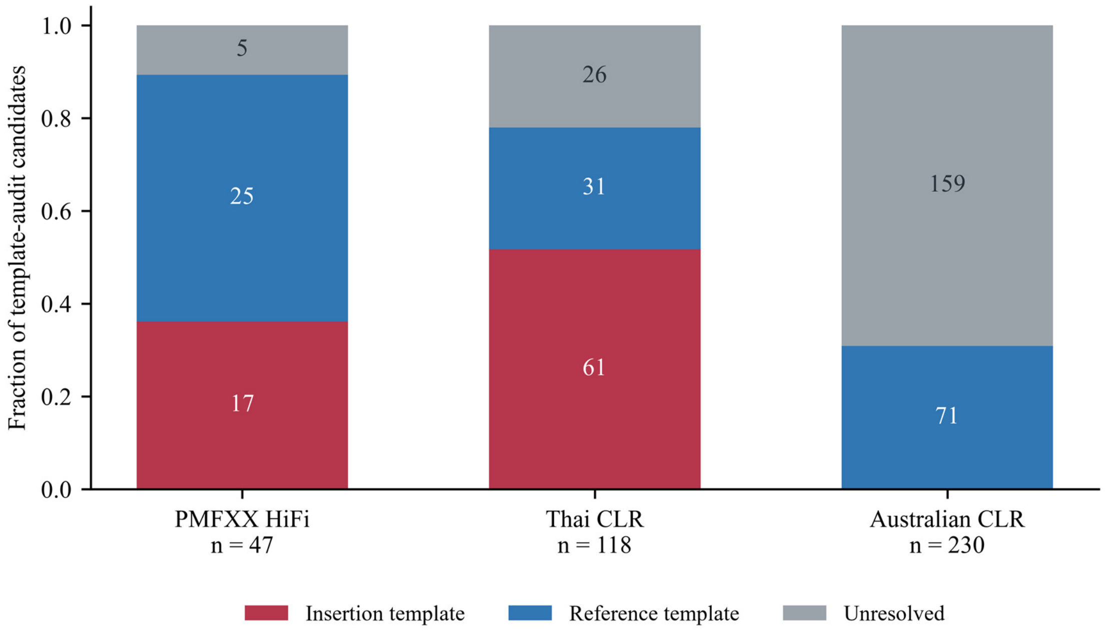

Figure S3. Symmetric local-template sensitivity analysis. Insertion-template, reference-template and unresolved calls among 47 PMFXX HiFi, 118 Thai CLR and 230 Australian CLR candidates. This subset uses different eligibility criteria from the structural and allele-context denominators in Figure 7.

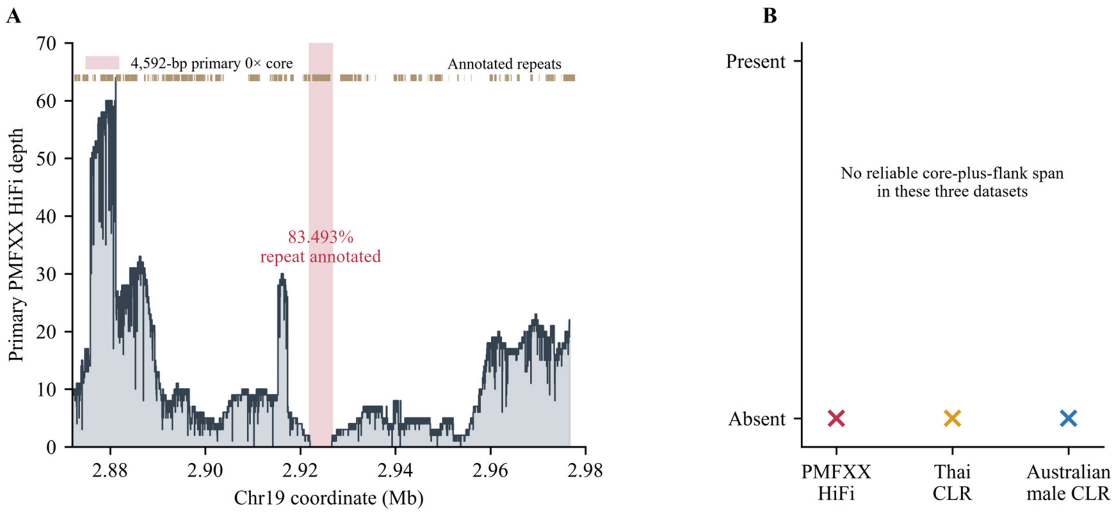

Figure S4. Mapping and repeat context around Gap3 outside the primary candidate SDR. A, PMFXX HiFi primary depth and repeat annotations surrounding the 4592-bp primary 0 × core (Chr19:2,922,034–2,926,625; shading); 83.493% of the core is repeat annotated. B, No reliable continuous core-plus-flank span was identified in the PMFXX HiFi, Thai CLR or Australian male CLR audits. In the family Illumina data at MAPQ ≥ 20, 22.1% of core positions were covered and core depth was 18.7% of adjacent-flank depth. These observations describe local mapping complexity without specifying a structural mechanism.

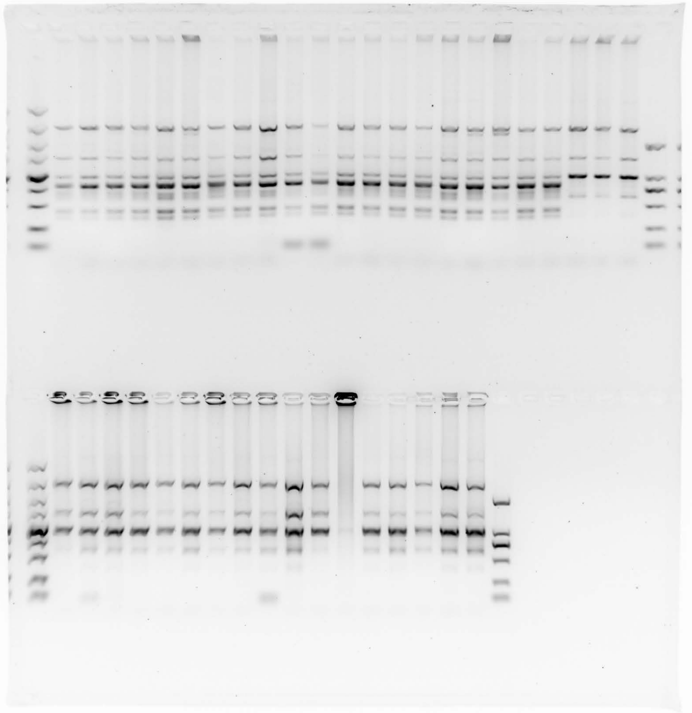

Figure S5. Uncropped source gel for Figure 4. The original image is reproduced without lane rearrangement, local intensity adjustment or added target-band boxes. Upper row: females 0-1 through 0-5 and 0-11 through 0-25, then males 1-1 through 1-3. Lower row: males 1-4, 1-5 and 1-11 through 1-25. Prefix 0 denotes female and 1 male. The left ladder is GL DNA Marker 5000 and the right ladder Trans2K DNA Marker. These are the 40 market-sourced animals shown in the main figure, not an additional cohort. The gel records PCR products, not independently sequence-confirmed INS54 genotypes for all lanes.

Table S1. Primary target and 44 non-target sequences used in the ONT specificity screen.

Coordinates are 1-based and inclusive on the PMFXX reference. The target was Chr19:2,680,000–2,920,000. All 44 decoys were non-target reference similarity hits of ≥500 bp and ≥80% identity, expanded by 2-kb flanks. Identity and alignment length refer to the best reference-similarity hit, not the candidate ONT read. The table reproduces the original manifest; it is also supplied as a machine-readable TSV.

Sequence	Reference coordinates	Identity	Aligned bp
TARGET	Chr19:2680000–2920000	1.00000	240001
DECOY_001	Chr02:37666944–37678314	0.83597	7383
DECOY_002	Chr03:34816765–34828106	0.84121	7343
DECOY_003	Chr04:40376138–40389057	0.82772	8997
DECOY_004	Chr05:24942153–24955478	0.86947	9400
DECOY_005	Chr06:8179355–8192081	0.90184	8934
DECOY_006	Chr06:30227091–30238425	0.84378	7336
DECOY_007	Chr07:33314866–33326214	0.83918	7350
DECOY_008	Chr08:35712802–35724159	0.83980	7372
DECOY_009	Chr09:1228549–1240852	0.85985	8926
DECOY_010	Chr09:48510958–48522327	0.83991	7371
DECOY_011	Chr13:17913147–17926043	0.92183	8981
DECOY_012	Chr13:40220353–40231668	0.92290	7393
DECOY_013	Chr14:18751726–18763075	0.83897	7365
DECOY_014	Chr14:39045505–39056837	0.83939	7353
DECOY_015	Chr15:12605639–12617003	0.83993	7378
DECOY_016	Chr15:20198259–20209594	0.84776	7337
DECOY_017	Chr17:47720076–47732346	0.88460	9064
DECOY_018	Chr18:1805554–1817223	0.91050	7810
DECOY_019	Chr19:3754870–3766555	0.83232	7735
DECOY_020	Chr23:23782021–23796543	0.80951	10657
DECOY_021	Chr24:34293508–34304862	0.83825	7357
DECOY_022	Chr26:15946998–15958354	0.83814	7358
DECOY_023	Chr26:31839319–31862321	0.87831	20525
DECOY_024	Chr26:37196001–37207308	0.89897	7503
DECOY_025	Chr29:24270399–24282438	0.92148	8164
DECOY_026	Chr30:796348–807607	0.85211	7262
DECOY_027	Chr30:41707414–41718795	0.83145	7416
DECOY_028	Chr31:25682931–25694312	0.83484	7393
DECOY_029	Chr32:17145245–17156615	0.83893	7382
DECOY_030	Chr32:24459844–24471181	0.84058	7339
DECOY_031	Chr33:19732344–19743700	0.83786	7358
DECOY_032	Chr34:2926063–2937400	0.84065	7355
DECOY_033	Chr34:16288389–16299745	0.83814	7358
DECOY_034	Chr35:558011–569384	0.83939	7378

Sequence	Reference coordinates	Identity	Aligned bp
DECOY_035	Chr35:37341232–37359982	0.83977	7358
DECOY_036	Chr36:34474393–34485739	0.84036	7348
DECOY_037	Chr37:1689651–1701031	0.83821	7386
DECOY_038	Chr38:7185195–7197947	0.93431	9073
DECOY_039	Chr38:29720129–29731485	0.83841	7358
DECOY_040	Chr39:9109129–9122858	0.88249	9846
DECOY_041	Chr39:15390405–15400917	0.91285	6885
DECOY_042	Chr40:12713686–12725050	0.83711	7379
DECOY_043	Chr41:13049933–13061287	0.83857	7359
DECOY_044	Contig10:240986–252341	0.83814	7358

Supplementary Method S1. Protein-level local alignment.

The original sequence-comparison script used a custom Smith–Waterman implementation in Python 3.11.9 with NumPy 1.26.4. Scores were +2 for a match, −1 for a mismatch and −2 per gap character (linear gap penalty). The dynamic-programming matrix was initialized at zero. Each cell selected the maximum of zero, diagonal match/mismatch, deletion and insertion; ties followed that order. Traceback began at the first strictly highest-scoring cell and ended at a zero-valued cell. Identity counted identical non-gap positions divided by the full gapped alignment length.

Reference gene24 and gene26/dmd-3 products were 366 and 514 amino acids, respectively. Their local alignment contained 308 columns and 269 identical positions (87.3377%, reported as 87.34%). The aligned intervals were gene24 residues 64–357 and gene26 residues 177–474. This is a local sequence-similarity measurement, not proof of transcription-factor activity or independently resolved W/Z proteins.

The supplied Supplementary_Method_S1_Protein_Alignment.py reproduces the original function. Protein FASTA inputs and Protein_Alignment_Result.json accompany it.
